# Derivation of porcine extraembryonic endoderm‐like cells from blastocysts

**DOI:** 10.1111/cpr.12782

**Published:** 2020-03-20

**Authors:** Yan Li, Shuang Wu, Yang Yu, Heng Zhang, Renyue Wei, Jiawei Lv, Mingming Cai, Xu Yang, Yu Zhang, Zhonghua Liu

**Affiliations:** ^1^ Laboratory of Embryo Biotechnology College of Life Science Northeast Agricultural University Harbin China; ^2^ School of Life Sciences Westlake University Hangzhou China

**Keywords:** extraembryonic endoderm cell, FGF, pig, primitive endoderm

## Abstract

**Objectives:**

Extraembryonic endoderm (XEN) cells are isolated from primitive endoderm (PrE) of blastocysts. Just like PrE, XEN cells have the ability to differentiate into parietal endoderm (PE) and visceral endoderm (VE), and therefore, they are useful tools for studying mechanisms of PrE cells development and differentiation. Pig is an ideal model for studying human cardiovascular and metabolic diseases and a potential organ source for allotransplantation, while no XEN cell has been obtained from porcine embryos.

**Materials and Methods:**

Using a serum‐free culture system, we directly derived porcine extraembryonic endoderm‐like cells (pXEN‐like cells) from day 6‐7 blastocysts, which could maintain self‐renewal for at least 30 passages.

**Results:**

The pXEN‐like cells resembled mouse XEN cells with large and flat clone morphology and expressed XEN marker genes but not pluripotent genes. Upon in vitro induction, the cells could differentiate into VE and PE. FGF/MEK signalling was not only essential for the maintenance of pXEN‐like cells, but also the induction of pXEN‐like cells from porcine embryonic stem (pES) cells.

**Conclusions:**

We directly obtained cell lines with XEN characteristics from porcine embryos for the first time. The cells will be helpful tools for studying embryonic development and cell differentiation, which also represent promising cell sources for human regenerative medicine.

## 1 INTRODUCTION

After the first germ layer differentiation, a fertilized egg develops into a blastocyst.^1^ Prior to implantation, the outer layer of blastocysts forms trophectoderm, which contributes to the placenta and foetal membrane, and the internal cells of blastocysts are called inner cell mass (ICM).^2^ And then, the ICM differentiates into epiblast (Epi) and primitive endoderm (PrE). The Epi contributes to the foetal body and the PrE segregates into two subpopulations of extraembryonic endoderm: visceral endoderm (VE) and parietal endoderm (PE).^3^, ^4^

Recently, researchers established different kinds of stem cells from different embryonic stages. Embryonic stem (ES) cells are derived from ICM or Epi, and they can be maintained in vitro in an undifferentiated state.^5^, ^6^ Trophoblast stem (TS) cells obtained from trophectoderm exhibit flat clonal morphology and express specific marker *Cdx2*.^2^ Extraembryonic endoderm (XEN) cells are isolated from PrE of the implanted blastocysts, and they have epiblast stem cell‐like clonal morphology. However, XEN cells express markers typical of extraembryonic endoderm derivatives (*Sox17, Gata4 and Gata6*), but not those of the epiblast (*Oct4, Sox2 and Nanog*) or trophoblast (*Cdx2*). Since XEN cells have the same ability to differentiate into PE and VE as PrE, they are useful tools for studying the mechanisms of PrE cells development and differentiation.^7^

As for mouse, we can get XEN cells via three different ways. First, the cells can be derived directly from embryos.^8^, ^9^ Second, they can be converted from embryonic stem (ES) cells.^10^ Third, they can be induced from fibroblasts by overexpression of pluripotent genes, which emerge during the transition of somatic state to pluripotent state.^11^ Like mouse, rat XEN cells can be derived from embryos directly,^12^ and canine XEN‐like cells are induced from embryonic fibroblasts by introducing pluripotent genes.^13^ Pigs are not only important farm animals, but also potential candidates as human disease models because of their similarities to humans in organ size and physiological characteristics.^14^ Porcine pluripotent stem cells are useful cell sources for fundamental study and generating animal models. During the last decade, a large number of porcine ES‐like cell lines were acquired,^15^, ^16^ while research on porcine XEN cells was few. Recently, Shen et al successfully derived porcine XEN cells (pXENCs) from porcine pluripotent stem (pPS) cells,^17^ while no report shows pXEN cells can be derived from embryos directly.

Mouse XEN cell lines, derived from the PrE of blastocysts, have unique characteristics.^7^ Previous studies showed that Fgfr2 was enriched in mouse PrE cells.^18^ In addition, the studies of intracellular signal transduction suggested that FGF/ERK signalling was a critical pathway to segregate PrE from epiblast.^19^, ^20^ In the study of mouse XEN cells, some results showed that the maintenance of XEN cells in vitro also needs FGF/ERK signalling activation, and during the differentiation of mouse ES cells into XEN cells, FGF/ERK signalling is also required.^10^ But as for rat XEN cells, their proliferation is dependent on LIF signalling.^21^ Thus, the signalling pathways that regulate the growth of XEN cells have species specificity.

Here, using a serum‐free culture system, we derived porcine extraembryonic endoderm‐like cells (pXEN‐like cells) from day 6‐7 blastocysts. The cells expressed XEN marker genes *Gata4*, *Gata6* and *Sox17* and could differentiate into VE and PE upon induction. The maintenance of pXEN‐like cells depended on bFGF instead of LIF, and bFGF addition could induce pXEN‐like cells from porcine ES cells. The pXEN‐like cells will be a helpful tool for studying porcine embryonic development and represent a promising cell sources for contrasting human disease models.

## 2 MATERIALS AND METHODS

### 2.1 Animal

Porcine ovaries were collected from Guanglin slaughterhouse, and porcine spermatozoa were from Hongfu Pig Farm. All experiments involving animals were approved and conducted according to the guidelines of the Laboratory Animal Ethics Committee of Northeast Agricultural University, China.

### 2.2 Production of porcine blastocysts

Porcine blastocysts were got by in vitro oocyte maturation (IVM) and in vitro fertilization (IVF) as previously reported.^16^ Briefly, after 42 hours of cultivation in the mature medium, we selected high‐quality oocytes with the first polar body for IVF.^22^ Then, the fertilized embryos were cultured in porcine zygote 3 (PZM‐3) medium at 39°C in 5% CO_2_ atmosphere for 6‐7 days.

### 2.3 Derivation and culture of pXEN‐like cells

Porcine expanded blastocysts were selected to establish pXEN‐like cells. After mechanically removing zona pellucida using glass pipettes, the embryos were seeded on mitomycin‐treated mouse embryonic fibroblasts and cultured with PXEN medium. PEXN medium consisted of 47.5% knockout Dulbecco's modified Eagle's medium (DMEM), 0.5% B27, 0.25% N2, 0.1 mmol/L β‐mercaptoethanol, 1% MEM non‐essential amino acids, 1% penicillin‐streptomycin, 2 mmol/L l‐glutamine, 0.5 mg/mL BSA, 1 ng/mL hLIF and 16 ng/mL bFGF. After 3‐5 days, the outgrowths expanded from the edge of the embryos, and they were mechanically isolated from the feeder cells. The outgrowths tore into small pieces and transferred onto fresh feeder cells for subculture. pXEN‐like cells were passaged every 4‐5 days using 1 mg/mL collagenase IV and were cultured in humidified conditions with 5% O_2_, 5% CO_2_ and 90% N_2_ at 39°C.

The small molecule inhibitor PD0325901 (Stemgent, 04‐0006) was used at the following concentrations: 0.6 μmol/L, 3 μmol/L or 15 μmol/L.

### 2.4 Culture of porcine pluripotent stem cells

pES cells were derived from porcine embryos and maintained on mitomycin‐treated mouse embryonic fibroblasts. EPSCM medium was used to culture pES cells, which was changed daily. The composition of EPSCM medium is consistent with previous publication.^23^

The porcine induced pluripotent stem (piPS) cells are from Pengtao Liu's group by a gift, which was cultured in pEPSCM medium. The medium was changed every day.^24^

### 2.5 Immunofluorescence staining

The samples were fixed with 4% (w/v) paraformaldehyde (PFA) for 30 minutes at room temperature and then were permeabilized in 1% (v/v) Triton X‐100 for 1 hour. After blockage at 37°C for 1 hour with 1% (w/v) BSA, the cells and embryos were incubated with primary antibodies for SOX2 (Santa Cruz, sc‐17320), NANOG (Pepro Tech, 500‐P236), OCT4 (Santa Cruz, sc‐8628), CDX2 (Biogenex, MU392A‐UC), GATA4 (Abcam ab81598), GATA6 (Abcam, ab22600) or AFP (Abnova, H0000174‐M01) at 4°C overnight. After thoroughly washing, the corresponding secondary antibodies were added in and incubated at 37°C for 1 hour. Before examination, the nuclei were stained with Hoechst 33342 (Sigma, B2261).

### 2.6 RNA isolation and qPCR

Total RNA was extracted according to manufacturer's instruction using RNeasy Mini Kit (Qiagen). cDNA was synthesized using High‐Capacity cDNA Reverse Transcription Kit (Applied Biosystems, 4368814). Quantitative real‐time PCR was performed using Premix ExTaqTM (Perfect Real Time, TaKaRa, RR420A) and 7500 Real‐Time PCR System (Applied Biosystems). All the used primers are listed in Table 1.

**Table 1:** Primers for Quantitative real‐time PCR

Gene name	Forward primers	Reverse primers
*Oct4*	CAAACTGAGGTGCCTGCCCTTC	ATTGAACTTCACCTTCCCTCCAACC
*Sox2*	CATCAACGGTACACTGCCTCTC	ACTCTCCTCCCATTTCCCTCTTT
*Nanog*	AATGATCGTCACATATCTTCAGGCTGTA	GTTCCATGGGCTCAGTGGTCAAG
*Lif*	CACTGGAAACACGGGGCA	CACTGGAAACACGGGGCA
*Lifr*	CTCATCCCAgTGGCAGTG	CCAGAACCTCAACATTAT
*Gp130*	GACCGTTGATTATTCTCCTGTGT	TATGTGGTGGATTGGGCTTC
*Jak1*	TCGGTATGGCGGCATTCTC	CTGCTCCGTCTTGGGGTCT
*Stat3*	AACTTTATCAGTAAGGAGAGGGA	GACACAAGGATGTTGGTAGCA
*Fgf2*	GCGACCCTCACATCAAACT	CAGTGCCACATACCAACT
*Fgfr1*	ACTGCTGGAGTTAATACCACCG	GCAGAGTGATGGGAGAGTCC
*Fgfr2*	TGATGATGAGAGACTGTTGGCATGC	TCCAAGTAGTCCTCATTGGTCGTG
*Mek*	CGAGACAGCCCAGACAACAA	CGCATACCCAACAAGCAAATA
*Gata4*	CGACACCCTAATCTCGATATGTT	TTCATCTTGTGGTAGAGGCCG
*Gata6*	TTGGTTATTCCCGAATTTCTCCG	CATTCCTGCAAACTGGGTGATAC
*Sox17*	GACATGAAGATGAAGGGCGA	GTACTTGTAGTTGGGGTGGTCC
*Cdx2*	GCTATAAATGCCAGAGCCAACC	AACAACCCAAACAGCAGCAAC
*Socs3*	CCCCCCTAGAAGAGCCTATT	CCGTTGACTGTTTTCCGACA
*Gapdh*	GCAAAGTGGACATTGTCGCCATCA	TCCTGGAAGATGGTGATGGCCTTT
*Snail*	CCGAGAGTTATGCTGCCTT	AGCGTGTGGCTTCTGATGT
*Sparc*	GCTCTCGCCTAAACCCAGTT	ACCCCTGTCGGATGTAGTGA
*Fox2a,*	GTATGCTGGGAGCGGTGAAG	GTAAGTGTTCATCCCGTTCATCC
*Hnf4a*	TGGTGGACAAAGACAAGAGGAAC	TGCTATCCTCGTAGCTTGACCTG
*Afp*	TGAGGCCGTCATTGCAGATT	TCAGTGCTGGACCCTCTTCT

### 2.7 Construction of TE‐labelled EGFP embryos

The lenti‐EGFP plasmid was a gift from Prof. Jiaqiang Wang (Northeast Agricultural University). Briefly, EGFP gene was promoted by elongation factor 1α short promoter and cloned into a lentiviral backbone. 293T cells were transfected with 24 μg of plasmids, 48 μL of LTX and 24 μL of PLUS regents, and the proportions of PMD2.G (Addgene#12259), PSPAX (Addgene#12260) and lenti‐EGFP were 1:2:3. The supernatants were collected at 24 hours and 48 hours after transfection, filtered by 0.45 μm filters and concentrated by centrifugal filters (Millipore) at 4°C and 4000 *g* for 30 minutes.

The 5.5 days of small cavity blastocysts were collected, and their zona pellucida was removed by Protease K (Promega, V302B). Then, the embryos were incubated in PZM‐3 medium containing lentivirus carrying EGFP gene. After 3 hours, the embryos were washed 3‐4 times with washing solution, and then, the infected embryos were cultured in PZM‐3 medium at 39°C in 5% CO_2_ atmosphere for 12‐24 hours.

### 2.8 Isolation and culture of trophectoderm

We isolated trophectoderm from day 6‐7 embryos transfected with EGFP gene using syringe needles (Figure S1), and then, the TE was seeded on feeders and cultured with PXEN medium. The medium was changed every day.

### 2.9 Embryoid body formation

piPS cells and pXEN‐like cells were digested into single cells and suspended in substrate‐free culture dishes with 15% FBS medium. The medium was changed every other day. After 3‐5 days of cultivation, embryoid bodies (EBs) formed.

### 2.10 VE and PE differentiation

When pXEN‐like cells reached 80% confluency, they were passaged with collagenase IV and seeded on Matrigel (Corning, 35423). The culture medium was changed to PXEN without bFGF and LIF but supplemented with 10 ng/mL BMP4 or 2 mM dorsomorphin (DM). After 5 days, VE and PE differentiation ability was evaluated.

### 2.11 Trypan Blue staining

Cells were digested into single cells and washed two times with DPBS. After centrifugation, the cells were resuspended and mixed with 0.4% Trypan Blue solution at a ratio of 9:1 in volume. After dyeing at room temperature for 3‐5 minutes, the numbers of live and dead cells were counted under the microscope.

### 2.12 RNA sequencing and analysis

Cells were washed 3 times with PBS and put in small centrifuge tubes packed with lysate for RNA sequencing. The sequencing reads were aligned to pig genome (Sscrofa10.2.87) using Tophat2 alignment software with default. Gene expression level was measured as fragments per kilobase million (FPKM). Gene differential expression was analysed using the DESeq2 package in R software. Heatmaps were generated using the heatmap package in R software. Pathway analysis was performed using DAVID (gene‐enrichment analysis using EASE Score). RNA sequencing raw data was uploaded to the National Center for Biotechnology Information database under the accession number GSE140414.

### 2.13 Statistical analysis

Statistical analysis was performed using SPSS 10.0.1. To determine the significance between groups, ANOVA was used. The data were presented as mean ± SD *P* < .05 was considered statistically significant (*), and *P* < .01 was considered extremely significant (**).

## 3 RESULTS

### 3.1 Derivation of pXEN‐like cells from blastocysts

**Figure 1:**
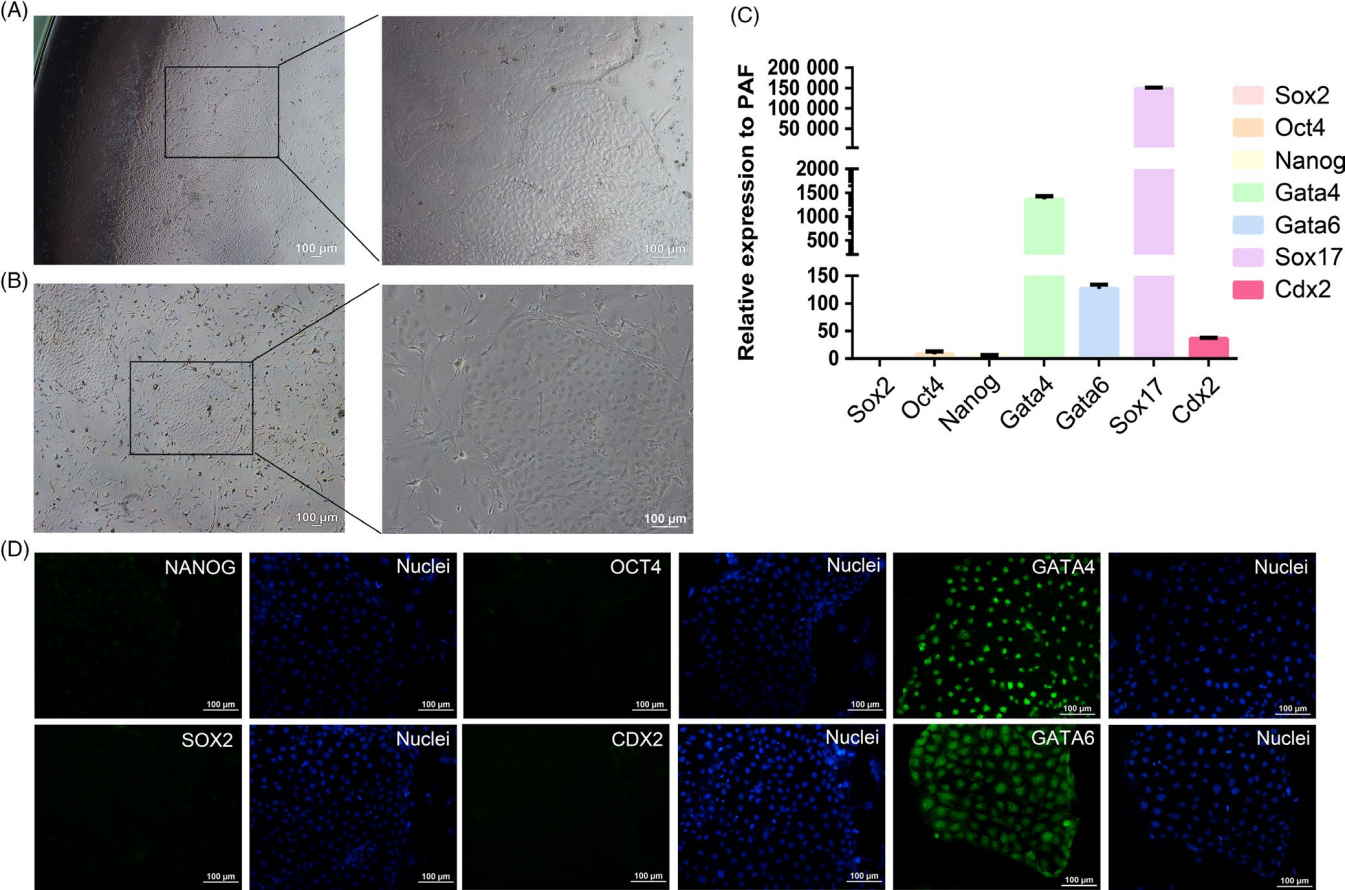
Derivation and characterization of pXEN‐like cells. A, A typical image of the outgrowth. B, The cellular morphology of the fifth passage. C, Expression levels of *Nanog*, *Oct4*, *Sox2*, *Gata4*, *Gata6* and *Sox17* were evaluated by quantitative RT‐PCR. Expression levels of the genes were relative to those of porcine adult fibroblasts (PAF). D, Immunofluorescence of pXEN‐like cells. The cells were positive for XEN markers GATA4, GATA6, but negative for pluripotent markers NANOG, OCT4 and SOX2 and TE marker CDX2 (green). Nuclei were stained with Hoechst 33342 (blue)

Day 6‐7 porcine blastocysts were cultured in PXEN medium, and 3‐5 days later, the outgrowth with cubical cells appeared (Figure 1A, Table 2). Then, the outgrowth was mechanically isolated and seeded on fresh feeder cells. The stably passaged cells were named pXEN‐like cells. They could be maintained at least 30 passages in vitro. The morphology of pXEN‐like cells was large and flat, just like that of mouse XEN cells (Figure 1B). Real‐time PCR and immunofluorescence analyses showed that pXEN‐like cells were positive for XEN markers, including *Gata4*, *Gata6* and *Sox17*, but not for pluripotent markers *Oct4*, *Sox2* and *Nanog* or TE marker *Cdx2* (Figure 1C‐D).

**Table 2:** Effect of bFGF on the establishment of pXEN‐like cells

Treatments	No. of embryos	Attached embryos (%)	Outgrowth (%)	Stable passage (%)
PXEN	52	32 (61.5 ± 12.4)a	14 (43.7 ± 11.5)a	10 (71.4 ± 1.8)a
PXEN‐bFGF	60	24 (40.0 ± 7.8)a	1 (4.2 ± 8.1)b	0b
PXEN + 3 μmol/L PD	46	16 (34.1 ± 7.1)a	0b	0b

Note

The experiment was repeated three times. Different superscripts in the same column represented significant differences (*P* < .05).

### 3.2 pXEN‐like cells are derived from ICM but not TE

**Figure 2:**
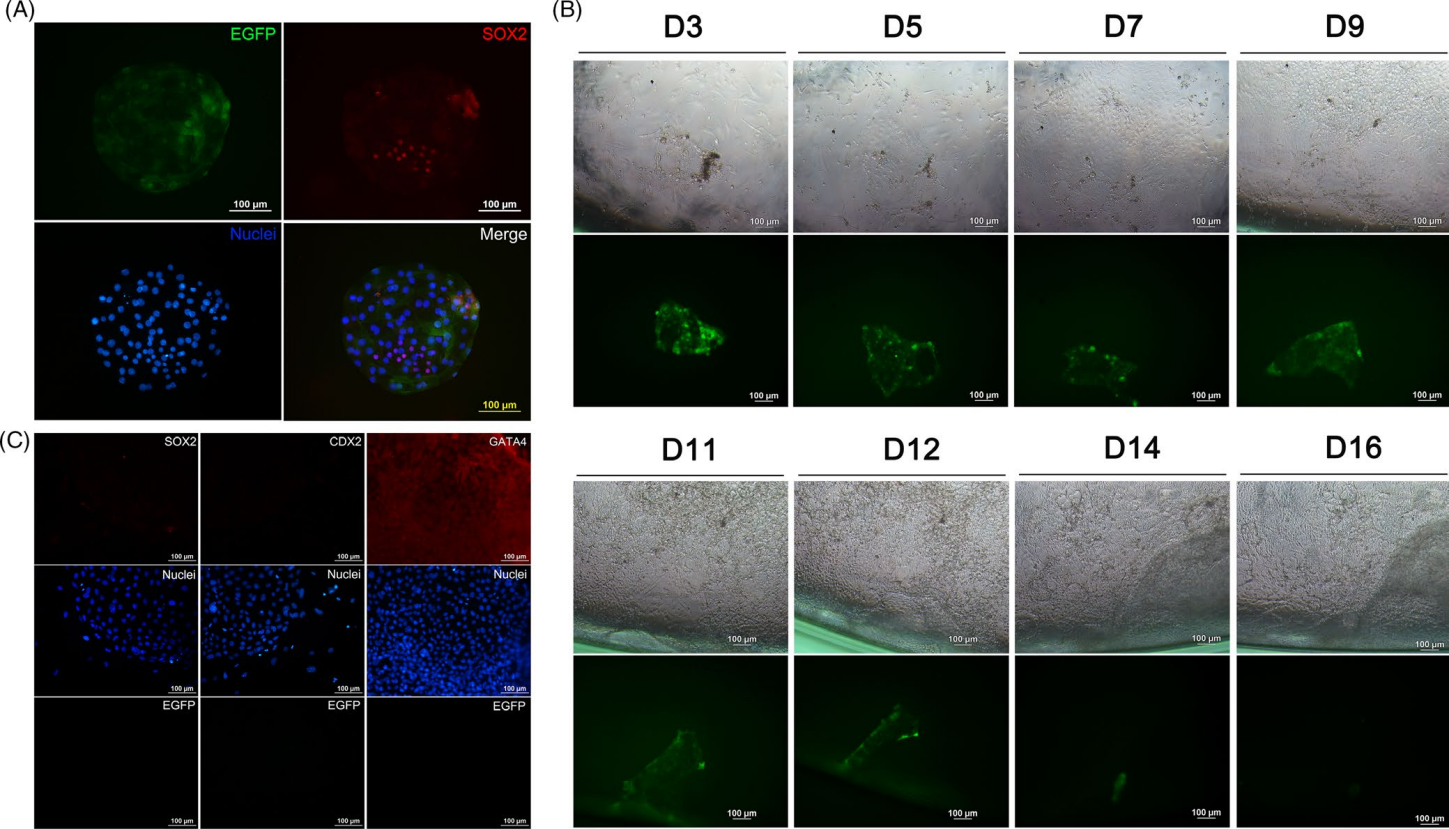
pXEN‐like cells are derived from ICM but not TE. A, The EGFP‐labelled TE cells (green) were negative for SOX2 (red). Nuclei were stained with Hoechst 33342 (blue). B, Outgrowth of the embryo inoculated for 16 days. C, Immunofluorescence staining of the derived cells. The cells were positive for GATA4 but negative for SOX2 and CDX2 (red). Nuclei were stained with Hoechst 33342 (blue)

Generally, XEN cells are derived from the PrE of the ICM of blastocysts.^8^, ^13^ To explore the source of pXEN‐like cells, we used TE‐labelled EGFP embryos for cell derivation (Figure 2A). After 16 days of cultivation, the EGFP‐labelled TE cells disappeared (Figure 2B). When we separated TE cells from the embryos and cultured them alone, they did not survive more than 7 days (Figure S1). Immunofluorescence staining showed that the obtained cells expressed PrE marker GATA4, but not the epiblast marker SOX2 or trophoblast marker CDX2 (Figure 2C). These results confirmed that pXEN‐like cells were derived from ICM but not TE.

### 3.3 pXEN‐like cells undergo PE and VE differentiation

**Figure 3:**
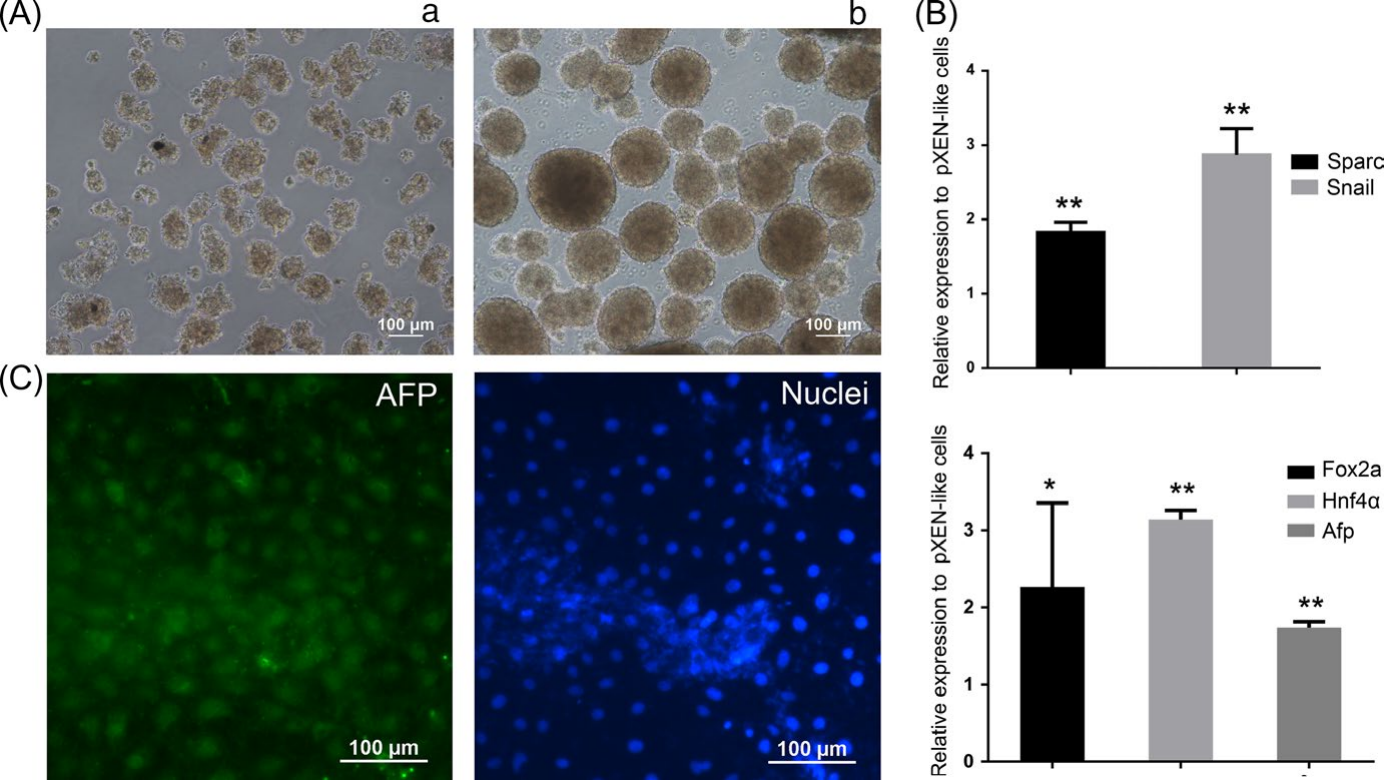
Differentiation capacity of pXEN‐like cells. A, Images of embryoid bodies of pXEN‐like cells (a) and piPS cells (b). B, Expression levels of PE markers *Sparc* and *Snail* and VE markers *Fox2a*, *Hnf4a* and *Afp* were evaluated by quantitative RT‐PCR. Expression levels of the genes were relative to those of pXEN‐like cells. C, Immunostaining for AFP (green). Nuclei were stained with Hoechst 33342 (blue)

In low‐adherent culture condition, the ES cells aggregate and form EBs, which recapitulate important aspects of early embryogenesis.^10^, ^25^ Using piPS cells, we successfully obtained EBs (Figure 3Ab). But pXEN‐like cells were difficult to generate EBs under the same condition (Figure 3Aa). Previous studies showed that XEN cells can differentiate into VE and PE of the yolk sac.^13^, ^17^ So, we used DM and BMP4 to treat the cells for PE and VE induction.^26^, ^27^ Five days later, we detected the expression pattern of PE marker genes *Sparc* and *Snail*, and VE marker genes *Fox2a*, *Hnf4a* and *Afp*. The result showed that *Sparc* and *Snail* were upregulated in the DM‐treated group, while *Fox2a*, *Hnf4a* and *Afp* were upregulated in the BMP4‐treated group (Figure 3B). Considering some studies showing that PE is an intermediate state which will be ultimately reprogrammed to VE,^26^ we examined the expression of VE marker AFP by immunofluorescence staining. The result showed that spontaneously differentiated cells were positive for AFP (Figure 3C). Taken together, we concluded that the pXEN‐like cells had the capacity to differentiate into VE and PE.

### 3.4 RNA‐seq analysis of pXEN‐like cells

**Figure 4:**
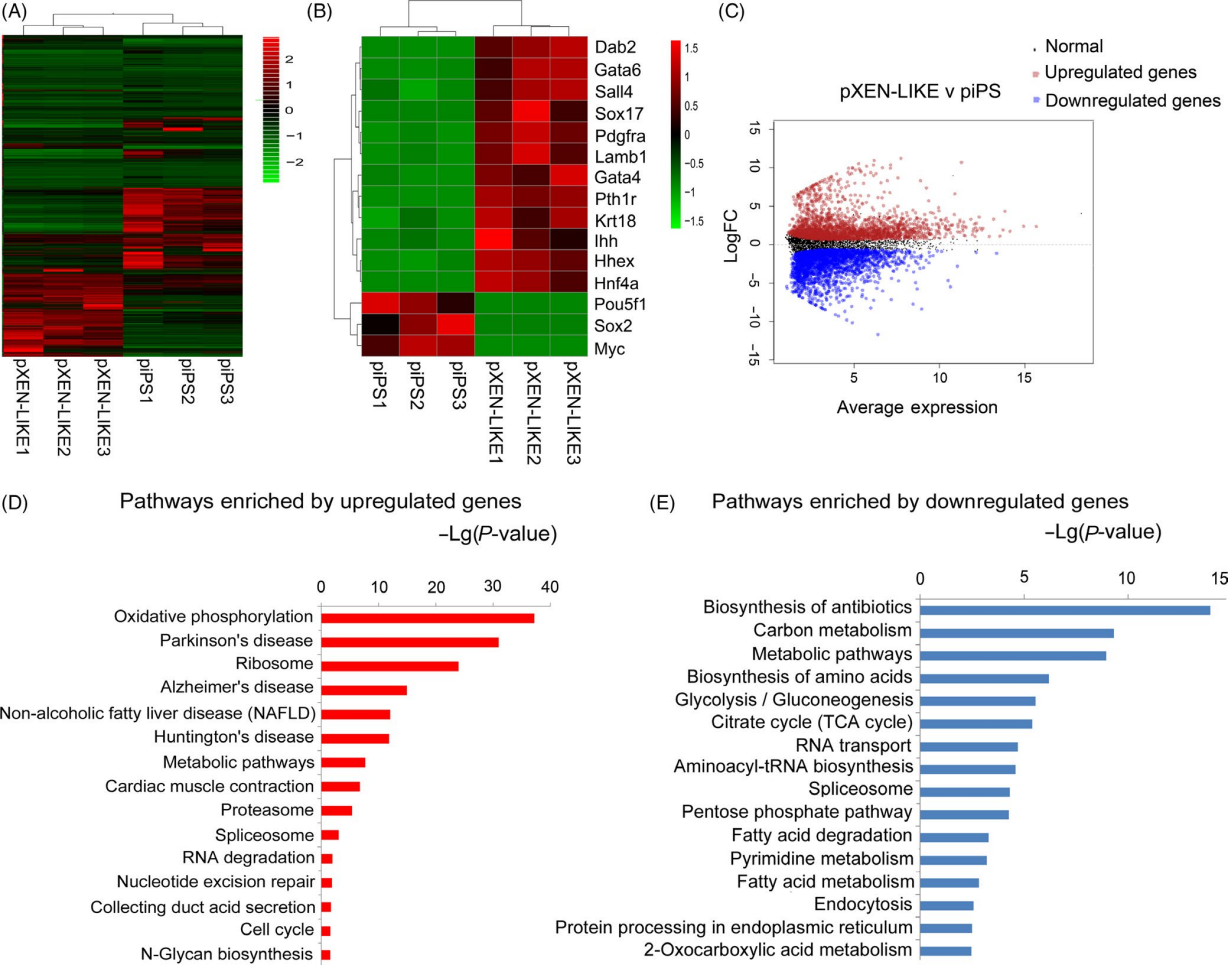
RNA‐seq analysis of pXEN‐like cells and piPS cells. A, Hierarchical cluster of transcriptome data using Spearman's correlation coefficient as a measure of distance between columns. B, Heatmap of gene expression levels for pXEN‐like cells and piPS cells. C, Volcano plot showing the differential expressed genes between pXEN‐like cells and piPS cells. D, Signalling pathway enrichment in upregulated genes of pXEN‐like cells compared with piPS cells. E, Signalling pathway enrichment in downregulated genes of pXEN‐like cells compared with piPS cells

Multi‐sample cluster analysis of the RNA‐seq data indicated highly reproducible gene expression patterns in piPS cells or pXEN‐like cells; however, the gene expression patterns of these two cell lines were significantly different (Figure 4A). pXEN‐like cells expressed typical XEN marker genes, but lacking pluripotent marker genes, such as *Pou5f1*, *Sox2* and *C‐myc* (Figure 4B). Compared with piPS, pXEN‐like cells had 1776 upregulated genes and 1988 downregulated genes (Figure 4C, Tables S2 and S3). Signalling pathway analysis showed that the upregulated genes were mostly enriched in disease related bioprocess, while the downregulated genes were mostly enriched in biosynthesis and metabolism relation (Figure 4D‐E).

### 3.5 Both derivation and maintenance of pXEN‐like cells are dependent on FGF/MEK signalling

**Figure 5:**
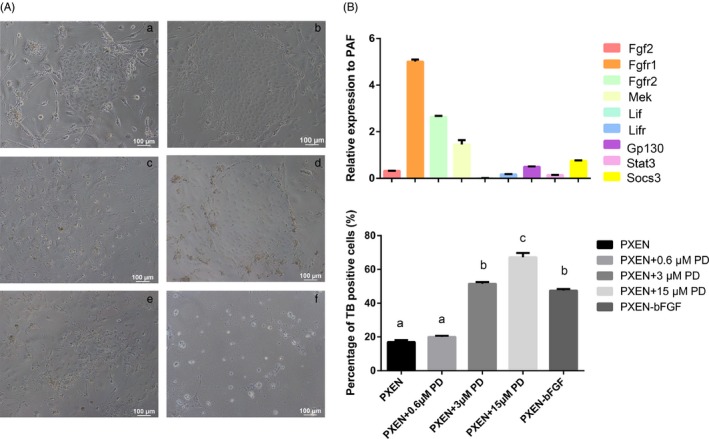
pXEN‐like cells are dependent on FGF/MEK signalling. A, pXEN‐like cells culture in PXEN (a), PXEN‐LIF (b), PXEN‐bFGF (c), PXEN + 0.6 μmol/L PD (d), PXEN + 3 μmol/L PD (e) and PXEN + 15 μmol/L PD (f) media for 2 d. B, Quantitative RT‐PCR assay for LIF signalling related genes and FGF/MEK signalling related genes. Expression levels of the genes were relative to those of PAF. C, Cell viability was evaluated by Trypan Blue staining. Values with different superscripts were significantly different (*P* < .05)

Since the culture medium contained only two cytokines, LIF and bFGF, we studied their effects on the maintenance of pXEN‐like cells. The results showed that the cell viability was reduced in the absence of bFGF, but not LIF (Figure 5Aa‐C). Then, we tested the expression levels of genes involved in LIF and FGF signalling pathways. We found most of genes related to FGF/MEK signalling were upregulated, while all of LIF signalling related genes were downregulated (Figure 5B). Meanwhile, the XEN marker genes were slightly upregulated in the medium without LIF (Figure S2). To verify the effect of FGF/MEK signalling on the maintenance of pXEN‐like cells, we used the FGF/MEK signalling pathway inhibitor PD0325901 to treat the cells and then evaluated their viability. The result showed the percentage of Trypan Blue positive cells changed in a dose dependent manner on PD0325901 (Figure 5Ad‐F, C).

Then, we tried to derive new cell lines from porcine blastocysts using PXEN culture medium without bFGF or addition of PD0325901. As a result, neither of the culture system supported new cell derivation (Table 2). These results demonstrated that both derivation and maintenance of pXEN‐like cells were dependent on FGF/MEK signalling.

### 3.6 Conversion of pig embryonic stem cells to pXEN‐like cells by bFGF

**Figure 6:**
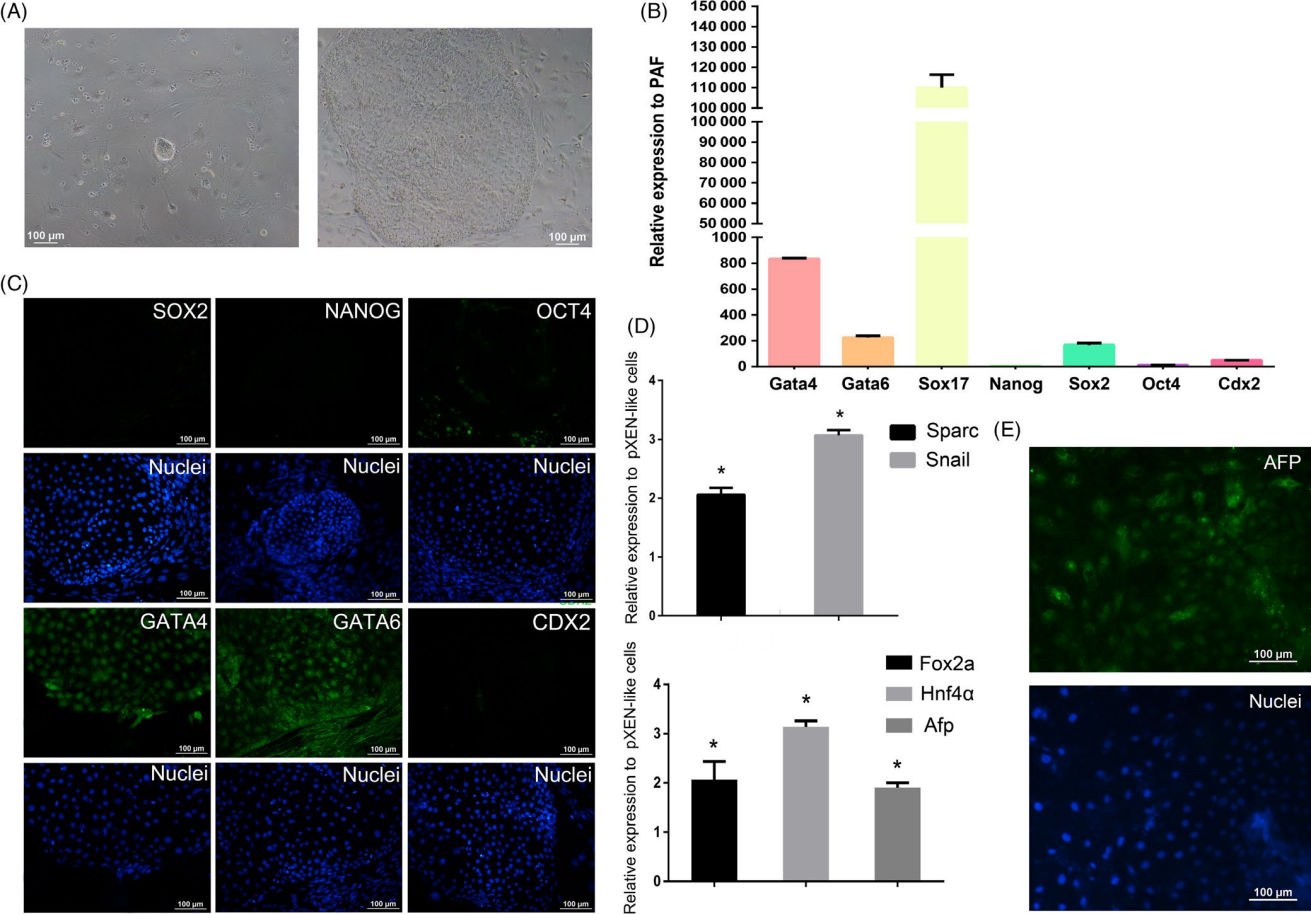
bFGF induce pES cells to turn to pXEN‐like cells. A, The morphology of the third passage of pES cells (left) and pXEN‐like cells (right). pES cells were treated with bFGF, and after 2 d, the clones changed to pXEN‐like morphology. B, Expression levels of *Nanog*, *Oct4*, *Sox2*, *Gata4*, *Gata6*, *Sox17* and *Cdx2* were evaluated by quantitative RT‐PCR. Expression levels of the genes were relative to those of PAF. C, Fluorescence analysis of pXEN‐like cells (green). Nuclei were stained with Hoechst 33342 (blue). D, Expression levels of PE marker (*Sparc* and *Snail*) and VE marker (*Fox2a*, *Hnf4a* and *Afp*) were evaluated by quantitative RT‐PCR. Expression levels of the genes were relative to those of pXEN‐like cells. E, Fluorescence analysis for AFP (green). Nuclei were stained with Hoechst 33342 (blue)

We then tried to establish pES cells with expanded pluripotency from porcine embryos using EPSCM as culture medium and finally obtained cells expressing pluripotent marker genes (Figure S3, Table S1). When we added bFGF to the medium, the cells exhibited XEN‐like clone morphology (Figure 6A) and expressed XEN marker genes *Gata4*, *Gata6* and *Sox17* but not pluripotent genes *Nanog*, *Oct4* and *Sox2* and TE marker gene *Cdx2* at both RNA and protein levels (Figure 6B‐C). Besides, these cells could differentiate into PE and VE upon appropriate induction (Figure 6D‐E). These results indicated that bFGF adding induced pESCs to turn to pXEN‐like cells.

## 4 DISCUSSION

The extraembryonic lineage of mammals is essential for the nutritive support of foetus and the patterning of early embryos As a major extraembryonic lineage, PrE is the secondary formed tissue during embryogenesis in mammals.^7^ XEN cells are a kind of stem cells isolated from the PrE, which have the same characteristics with PrE cells.^7^, ^8^

Here, we obtained pXEN‐like cells from day 6‐7 blastocysts in a serum‐free culture system. The pXEN‐like cells resembled mouse XEN cells with large and flat clone morphology. They expressed XEN marker genes *Gata4*, *Gata6* and *Sox17* but not pluripotent or TE markers. Like PrE, the pXEN‐like cells could differentiate into VE and PE upon induction. Compared with piPS cells, the pXEN‐like cells had 1,776 upregulated genes and 1988 downregulated genes, and FGF/MEK signalling was essential for their maintenance and induction.

Previous data showed that induced XEN cells rose in parallel to the iPS cells, indicating that pluripotent transcription factors drove cells to distinct cell fates during cell reprogramming.^11^ Meanwhile, we found two types of cells appeared at the beginning of porcine embryo inoculation for pluripotent cell derivation, one was compact and the other was flat. The cells with compact clonal morphology depend on high concentration of LIF for survival,^15^ and the flat ones were the pXEN‐like cells, depending on FGF/MEK signalling. Thus, we inferred that the two kinds of cells were from different cell types. As for mouse, naive ES cells required LIF/STAT3 signalling activation,^28^ and the data from single‐cell transcriptome sequencing of porcine early embryos showed that *Stat3* was also specifically expressed in ICM.^29^ At the same time, Shen et al found that activation of STAT3 could increase the cell number of ICM and the expression levels of pluripotent factors in porcine early blastocysts.^30^ So, LIF/STAT3 signalling pathway may be important for the authentic porcine ES cell derivation.

However, in mouse embryos, Fgfr2 is enriched in PrE cells, and embryos that lack ICM‐derived Fgf4 will affect the PrE development.^31^ As for mouse XEN cells, their maintenance needs FGF/ERK signalling activation, and FGF/ERK signalling is also required for mouse ESC differentiation to XEN cells.^8^, ^10^ In this study, our result confirmed that the pXEN‐like cells were also dependent on FGF/ERK signalling pathway. Therefore, we inferred that this pathway might be also important for porcine PrE development, although further experiment is needed. In addition, bFGF treatment could induce pXEN‐like cells in a pES cells culture condition. This indicated that inhibition of FGF/ERK signalling pathway may be favourable for the establishment of porcine ES cell lines.

As we know, the morphology of pXEN‐like cells is similar to primed state ES cells, which also depend on FGF signalling pathway. But the primed state ES cells derived from ICM^32^ or epiblast,^6^ expressed pluripotent markers such as *Oct4*, *Nanog* and *Sox2*. Additionally, these cells can differentiate into 3 germ layers in vitro and in vivo, just like the pES cells.^15^, ^16^, ^33^ However, the pXEN‐like cells expressed XEN marker genes but not pluripotent markers, and upon in vitro induction, they could only differentiate into extraembryonic endoderm.

In summary, we directly obtained cell lines with XEN cell characteristics from porcine blastocysts for the first time. The pXEN‐like cells are useful tools to study porcine embryo development and cell differentiation, which also represent a promising cell source for human regenerative medicine. Our study may also provide some clues for deriving authentic porcine ES cells.

## CONFLICT OF INTEREST

The authors declare no competing interests.

## AUTHOR CONTRIBUTIONS

Yan Li conceived the study, designed and carried out the experiments, and drafted the manuscript; Shuang Wu, Yang Yu, Heng Zhang, Renyue Wei, Jiawei Lv, Mingming Cai, Xu Yang carried out the experiments and participated in data analysis; Yu Zhang drafted and revised the manuscript; Zhonghua Liu supervised the study and supplied the funding. All authors have read, discussed and approved the final manuscript.

## Data Availability

RNA sequencing raw data was uploaded to the National Center for Biotechnology Information database under the accession number GSE140414. Other relevant data are within the manuscript and its Supporting Information files.
