# Supplementary figures and images for: Derivation of porcine extraembryonic endoderm‐like cells from blastocysts
Source: Cell Prolif. 2020 Mar 20;53(4):e12782. doi: 10.1111/cpr.12782 (PMC7162807; doi:10.1111/cpr.12782)

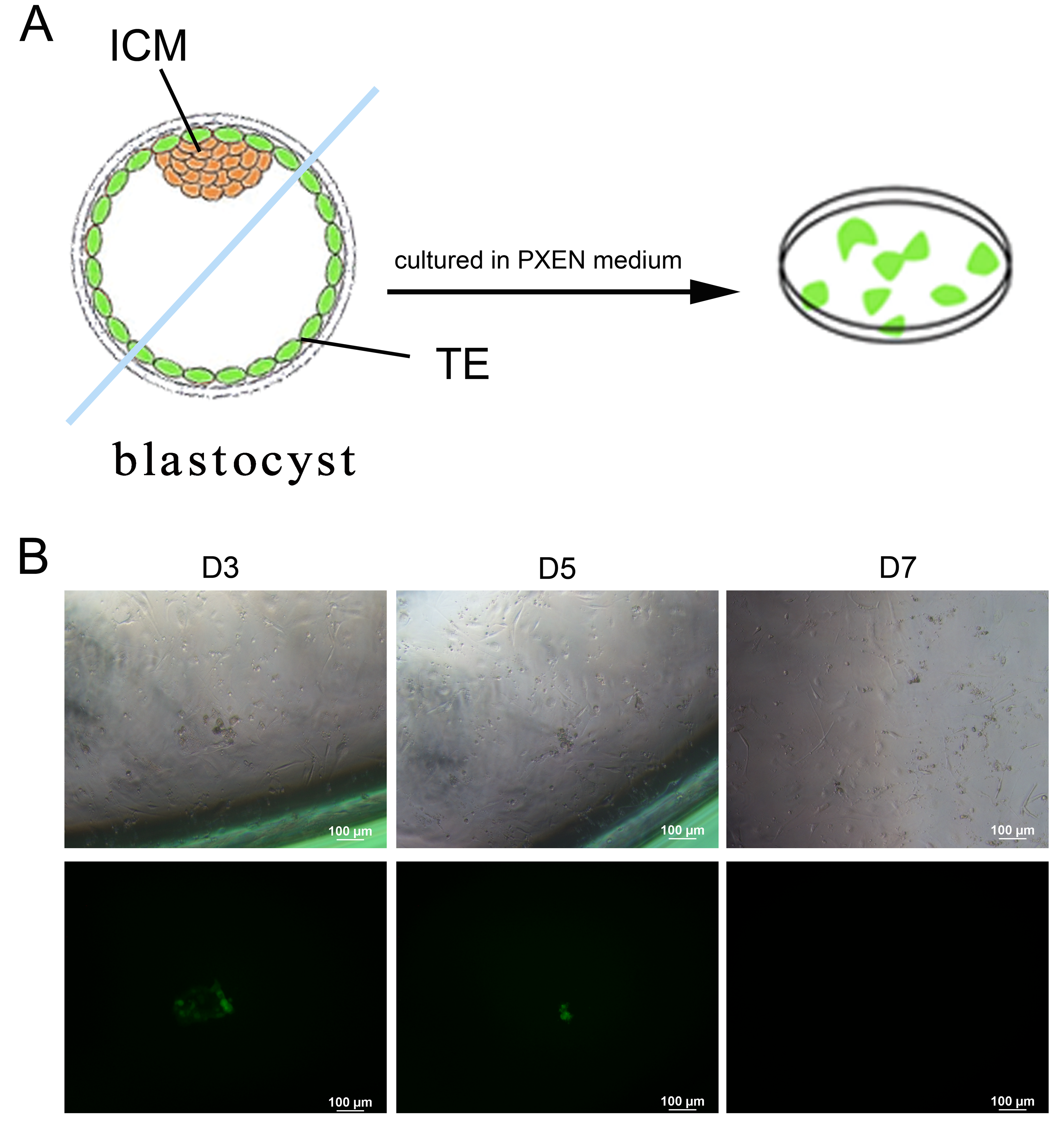

Supplement: Supplementary file 1 [file CPR-53-e12782-s001.tif]

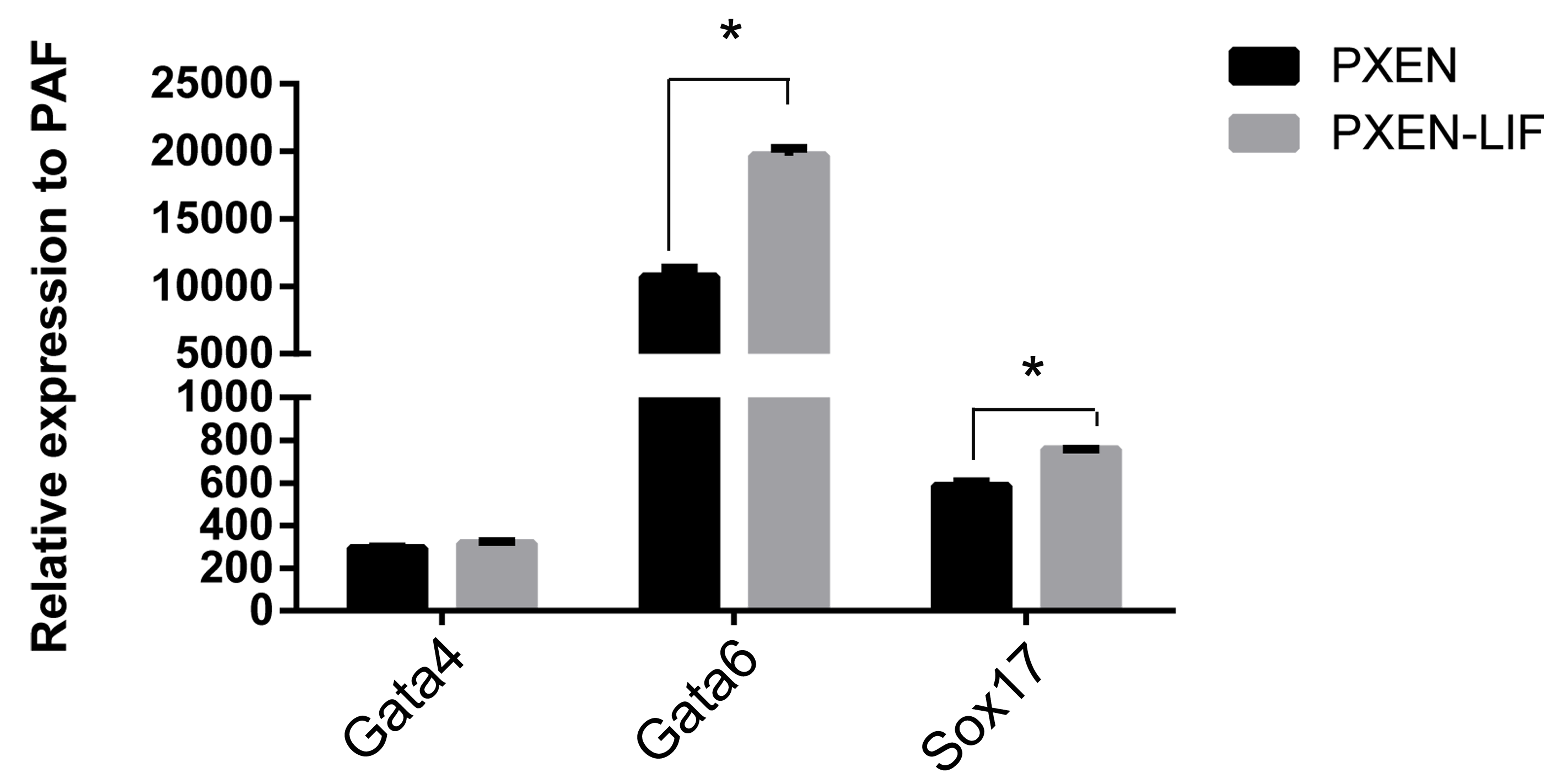

Supplement: Supplementary file 2 [file CPR-53-e12782-s002.tif]

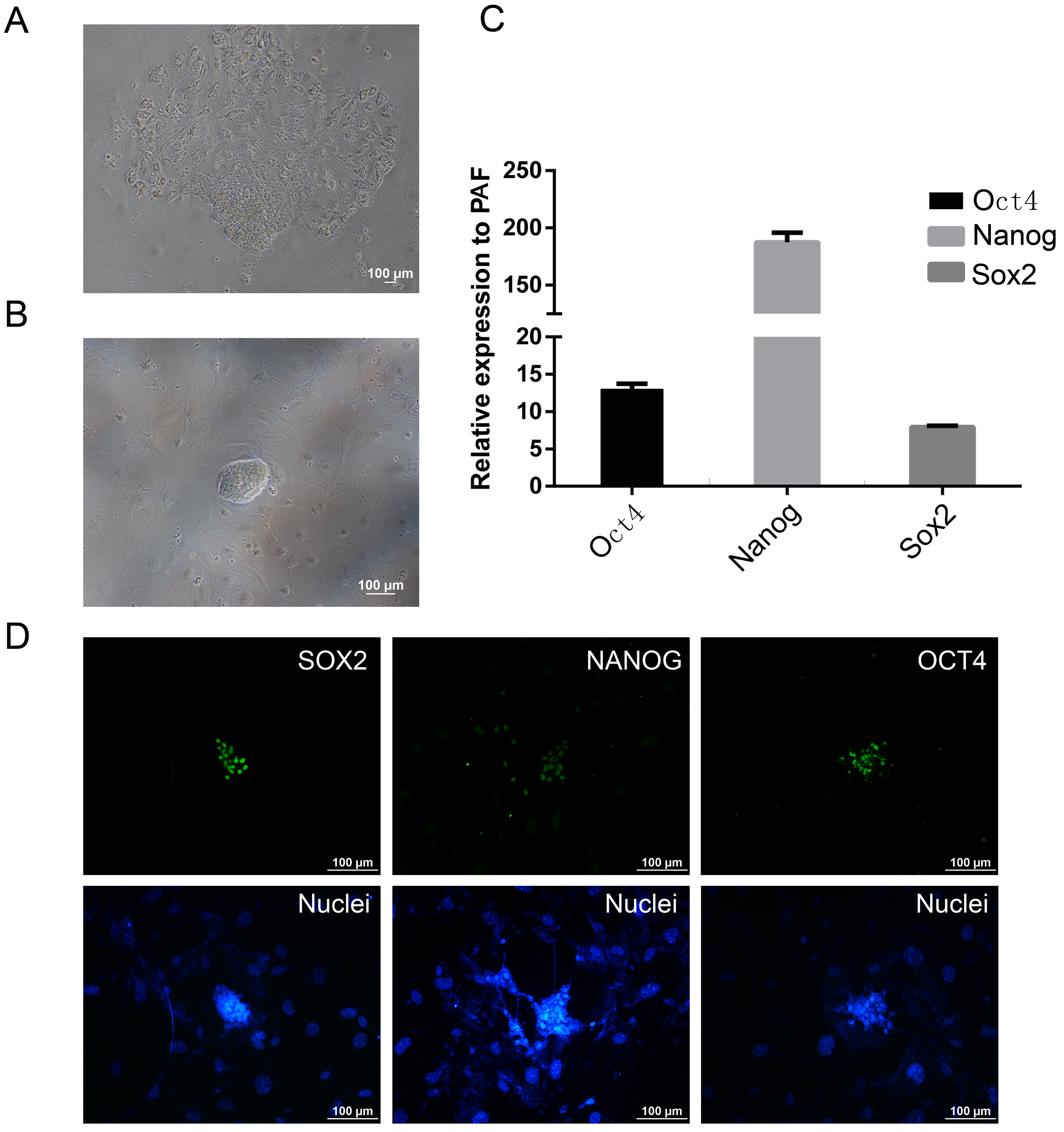

Supplement: Supplementary file 3 [file CPR-53-e12782-s003.tif]
