# Supplementary material for: Derivation of porcine extraembryonic endoderm‐like cells from blastocysts
Source: Cell Prolif. 2020 Mar 20;53(4):e12782. doi: 10.1111/cpr.12782 (PMC7162807; doi:10.1111/cpr.12782)
Supplement: Supplementary file 4 [file CPR-53-e12782-s004.docx]

**Table.S1 Establishment of pES cells in EPSCM medium**

| Medium | No. of Embryos | Attached embryos (%) | Outgrowth(%) | Stable passage(%) |
| --- | --- | --- | --- | --- |
| EPSCM | 41 | 20(48.7±1.1) | 5（25.0±6.7） | 1（20.0±14.1.） |

The experiment was repeated three times.

Different superscripts in the same column represented significant differences (P<0.05).
