# Supplementary material for: Derivation of porcine extraembryonic endoderm‐like cells from blastocysts
Source: Cell Prolif. 2020 Mar 20;53(4):e12782. doi: 10.1111/cpr.12782 (PMC7162807; doi:10.1111/cpr.12782)
Supplement: Supplementary file 7 [file CPR-53-e12782-s007.doc]

**Supporting Information**

**Supplementary Table**

**Table.S1 Establishment of pES cells in EPSCM medium**

**Table.S2 pXEN-like cells vs piPS cells up-regulated genes**

**Table.S3 pXEN-like cells vs piPS cells down-regulated genes**

**Supplementary Figure legends**

**Fig.S1 Isolation and culture of TE cells.** (A) Sketch map for TE isolation and culture. (B) The EGFP-labeled TE cells (green) were cultured for 7 days.

**Fig.S2 Expression levels of XEN marker genes were evaluated by quantitative RT-PCR.** Expression levels of the genes were relative to those of PAF.

**Fig.S3 Characterization of pES cells.** (A) A typical image of outgrowth in EPSCM. (B) The morphology of the third passage of pES cells. (C) Expression levels of *Nanog*, *Oct4* and *Sox2* were evaluated by quantitative RT-PCR. Expression levels of the genes were relative to those of PAF. (D) Fluorescence analysis of pluripotent makers NANOG, OCT4 and SOX2 in pES cells (green). Nuclei were stained with Hoechst 33342 (blue).
